# Phenotypic plasticity and the evolution of azole resistance in *Aspergillus fumigatus;* an expression profile of clinical isolates upon exposure to itraconazole

**DOI:** 10.1186/s12864-018-5255-z

**Published:** 2019-01-09

**Authors:** Margriet W. J. Hokken, Jan Zoll, Jordy P. M. Coolen, Bas J. Zwaan, Paul E. Verweij, Willem J. G. Melchers

**Affiliations:** 1 Department of Medical Microbiology, Radboud University Medical Center, Geert Grooteplein Zuid 10, 6525GA Nijmegen, the Netherlands; 2 Center of Expertise in Mycology Radboudumc/CWZ, Weg door Jonkerbos 100, 6532 SZ Nijmegen, the Netherlands; 3 Department of Plant Sciences, Laboratory of Genetics, Wageningen University, Droevendaalsesteeg 1, 6708PB Wageningen, The Netherlands

**Keywords:** *Aspergillus fumigatus*, Azole resistance, Phenotypic plasticity, Itraconazole, RNA-seq

## Abstract

**Background:**

The prevalence of azole resistance in clinical and environmental *Aspergillus fumigatus* isolates is rising over the past decades, but the molecular basis of the development of antifungal drug resistance is not well understood. This study focuses on the role of phenotypic plasticity in the evolution of azole resistance in *A. fumigatus*. When *A. fumigatus* is challenged with a new stressful environment, phenotypic plasticity may allow *A. fumigatus* to adjust their physiology to still enable growth and reproduction, therefore allowing the establishment of genetic adaptations through natural selection on the available variation in the mutational and recombinational gene pool. To investigate these short-term physiological adaptations, we conducted time series transcriptome analyses on three clinical *A. fumigatus* isolates, during incubation with itraconazole.

**Results:**

After analysis of expression patterns, we identified 3955, 3430, 1207, and 1101 differentially expressed genes (DEGs), after 30, 60, 120 and 240 min of incubation with itraconazole, respectively. We explored the general functions in these gene groups and we identified 186 genes that were differentially expressed during the whole time series. Additionally, we investigated expression patterns of potential novel drug-efflux transporters, genes involved in ergosterol and phospholipid biosynthesis, and the known MAPK proteins of *A. fumigatus*.

**Conclusions:**

Our data suggests that *A. fumigatus* adjusts its transcriptome quickly within 60 min of exposure to itraconazole. Further investigation of these short-term adaptive phenotypic plasticity mechanisms might enable us to understand how the direct response of *A. fumigatus* to itraconazole promotes survival of the fungus in the patient, before any “hard-wired” genetic mutations arise.

**Electronic supplementary material:**

The online version of this article (10.1186/s12864-018-5255-z) contains supplementary material, which is available to authorized users.

## Background

*Aspergillus fumigatus* is an ubiquitous fungus that plays an important role in carbon and nitrogen recycling in nature by degradation of organic biomass. It can be found in many terrestrial habitats, including soil and decaying organic matter [1]. A well-known example is the compost pile, which is considered a harsh environment as large numbers of microbes compete for nutrients, oxygen and space [2]. Various biological characteristics allow *A. fumigatus* to thrive in this environment, including rapid and efficient germination, tolerance for elevated temperatures, and a metabolism that is responsive to variation in nutrient sources [3, 4]. Furthermore, *A. fumigatus* propagates via asexual conidia that can be dispersed over wide geographic distances by air currents, and that can germinate to grow under a broad range of environmental conditions [5]. To survive and thrive, *Aspergillus* needs to rapidly adapt to these often challenging environments.

The remarkable ability of *A. fumigatus* to grow in diverse environments*,* contributes to its role as the predominant fungal pathogen of immunocompromised patients. As a result, *Aspergillus* infections are increasingly recognized as a global health problem. In humans, several diseases may be caused by *A. fumigatus,* ranging from allergic conditions to acute invasive aspergillosis [5, 6]. Azole antifungals are the primary choice for prevention and treatment of *Aspergillus* infections. Azoles inhibit the 14-α demethylase Cyp51A, as they function as a ligand of the heme group and interact with the protein structure through the azole drug side chains [7]. The inhibition of Cyp51A results in accumulation of toxic sterols, destabilisation of the plasma membrane and subsequent cell death [8]. However, *A. fumigatus* is able to adapt to the presence of azoles, which facilitates persistence of the species and compromises treatment options in the clinic [9].

Adaptation can be defined as the acquisition of adaptive traits through natural selection, which enables the organism to adjust to life in the prevailing or new environment [10]. Before natural selection occurs, the organism quickly adapts to a new environment by exerting its phenotypic plasticity (PP). PP allows organisms to adjust their physiology to adapt to a new and challenging environment, to still be able to maintain growth and reproduction [11, 12]. These adaptations precede the development of genetic changes and the resulting newly acquired traits. In filamentous fungi, these traits often arise by spontaneous mutation and subsequent selection if the new genotype has achieved a higher overall fitness through its phenotypic traits. Crucially, in *A. fumigatus*, this process has led to the development of azole resistance [13–16]. Azole resistant strains have emerged in patients with aspergilloma or chronic pulmonary aspergillosis during azole therapy, but also in for instance organic-waste that contained azole residues. Azole compounds are widely used for various applications including food production, plant protection, and material preservation [17]. Thus, any environment that contains azole compounds can result in azole resistance development in *A. fumigatus*, provided that the fungus can complete its life-cycle [15, 18, 19]. As the dominant mechanism of resistance (TR34/L98H) is suggested to have originated in the environment, various studies have focused on the role of azole use in agriculture in the development of azole-resistant isolates [20–22].

Previously, a lot of research focused on elucidating the genetic basis of azole resistance. The most common mechanisms of resistance in *A. fumigatus* are modifications in the *cyp51A* gene. Various non-synonymous mutations in this gene have been correlated to azole resistance [13, 23–25]. These mutations cause structural changes in the conserved active site, preventing azoles from binding to the enzyme [23]. It is not well understood how the fungus maintains fitness before these mutations arise, and knowledge of the molecular basis of this development is needed in order to assess the impact of this phenomenon.

To understand the development of resistance, we previously investigated whether genetic substitutions could be induced during exposure to azoles under laboratory conditions [9, 26]. Wild type *A. fumigatus* isolates were exposed to both medical and environmental azoles, or a mixture of these compounds. The induction experiments generally resulted in a resistant phenotype already within three passages. However, genetic alterations were only found after at least five passages. In three out of 12 clones of *A. fumigatus* cultured under itraconazole pressure, *cyp51A*-substitutions G138C or P216L were detected. These substitutions have been reported in patients who developed azole-resistant *Aspergillus* disease during itraconazole therapy [24, 25].

It is not well understood how the fungus maintains fitness before these mutations arise, and thus current knowledge suggests that adaptation follows a two-step process. Firstly, patient- and environmentally acquired resistance both require an actively reproducing fungus even when exposed to azole compounds. Therefore, PP adjusts the physiology of *A. fumigatus* to maintain growth and reproduction at least to some extent [27]. Secondly, after these physiological adjustments, the process of natural selection maintains the mutations from the gene pool that confer genetic resistance in the azole-containing environment. Clearly, it is important to understand the adaptation strategies of *A. fumigatus* in relation to azole resistance selection and the clinical implications thereof for management of *Aspergillus* diseases.

The capability of *A. fumigatus* to adapt in the human host or in the outside environment is an important fungal property, which contributes to treatment failure and to the increased numbers of resistant *A. fumigatus* found worldwide. Therefore, this report focuses on the role of PP in the evolution of azole resistance. For *A. fumigatus,* this plasticity may involve, for instance, increased efflux pump activity, the activation of several stress-response pathways, or altering its lipid metabolism as ergosterol depletion affects membrane homeostasis [28–31]. However, research is lacking on the cellular events that take place in the fungus directly after introduction of an antifungal compound to the environment. Furthermore, it is not known how the plasticity of the fungal physiology facilitates the selection of mutations. The early response is crucial, as the fungus needs to retain its metabolism and preferably its reproductive capability. In this study, the differential expression of the *A. fumigatus* genome was measured at various time points, directly after addition of itraconazole to the medium. The results provided important details on the initial transcriptional dynamics, and gave insights into membrane transporter activity, lipid metabolism, and MAPK cascade proteins, implicated to be involved in the process of phenotypic plasticity. These insights will allow us to find leads for targeted interventions.

## Results

We conducted an in vitro assay in which we exposed 24 h old mycelia of clinical *A. fumigatus* isolates grown at 37 °C, to sublethal concentrations of itraconazole which might permit gradual adaptation of the fungus to the new, stressful environment (Table 1). Strains 130–14, 147–03 and 155–40 were grown with 0.32 μl/mL, 0.15 μl/mL and 0.13 μl/mL respectively, corresponding to the IC50 concentrations for each strain which were determined by determining the dose-response curve for each strain (Additional file 1: Figure S1). Mycelia were then harvested after 30, 60, 120 or 240 min respectively, and relative expression was compared to the mycelia harvested directly after 24 h without the addition of itraconazole.

**Table 1:** Three clinical *A. fumigatus* isolates used for transcriptomic analysis in this study. The minimum inhibitory concentration (MIC) was determined for itraconazole (ICZ), voriconazole (VCZ) and posaconazole (PCZ)

		MIC (mg/liter)		
Isolate	Date of isolation	ITZ	VCZ	PCZ
130–14	25/11/2011	1	1	0.25
147–03	25/03/2013	0.5	1	0.25
155–40	23/10/2013	1	1	0.25

### Analysis of differentially expressed genes

**Fig. 1:**
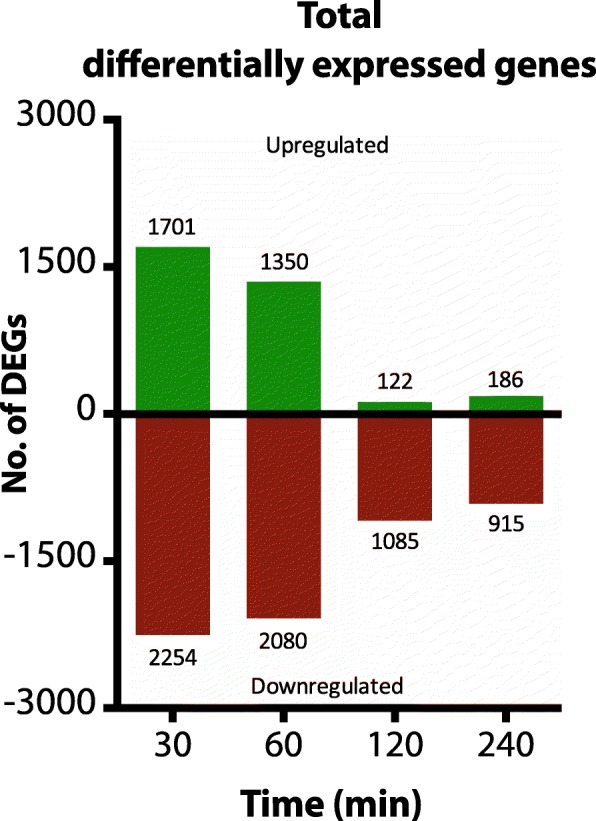
Graph representing the total numbers of differentially expressed genes after 30 min (3955 genes), 60 min (3430 genes), 120 min (1207 genes) and 240 min (1101 genes)

**Fig. 2:**
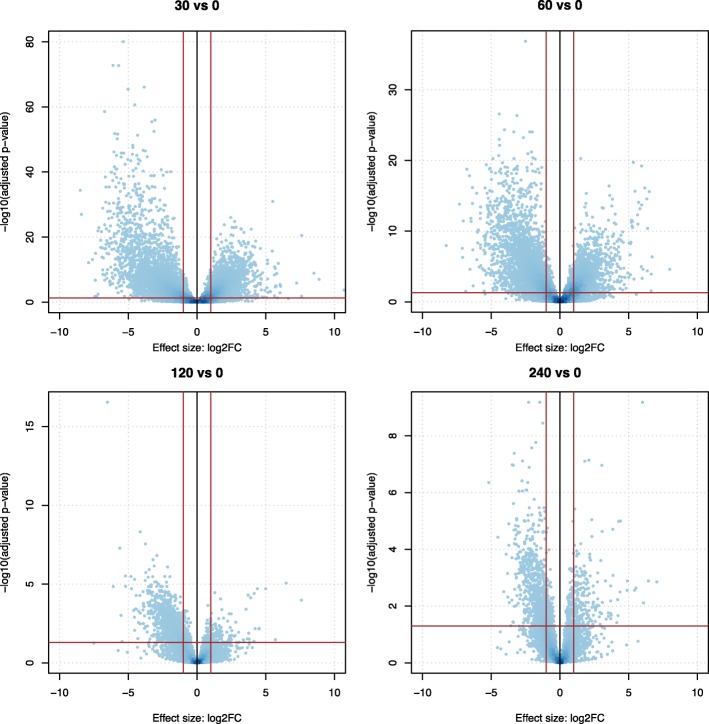
Volcano plots of the differentially expressed genes at all time points. For each gene, the log2FC value was plotted against its respective test *P*-value. The red line running parallel to the x-axis indicates the statistical cutoff (*P* < 0.05), whereas the red line running parallel to the y-axis indicates the biological cutoff (log2FC < − 1 or > 1). Note that the y-axis displays a variable –log10(adjusted *p*-value), as the number of significant genes decreases over time

A total of 401,706,537 sequence reads were obtained, with an average unique mapping percentage of 86.5% to the reference genome Af297 [32] (Additional file 2: Table S1). This implies that these isolates contain genomic sequences that are not present in the reference genome and thus cannot be mapped. Principle component analysis (PCA) revealed that the highest variation between samples was observed between the time points and that the replicates clustered together (Additional file 3: Figure S2). The samples of isolates 130–14 and 147–03 clustered together. The greatest amount of variation was found between the samples measured after 30 min and those measured after 240 min for isolate 130–14, or those measured after 0 min for isolate 147–03. The samples of isolate 155–40 were further removed from the other two isolates, although they show a similar pattern over time which could indicate a different basal expression level of this isolate. After comparing the gene expression between the samples taken without the addition of itraconazole (*t* = 0) and the samples incubated with itraconazole at the different time points, we identified 3955, 3430, 1207 and 1101 differentially expressed genes (DEGs) from a total of 9840 genes (Genome inventory, AF293 genome, AspGD, April 12, 2018) after 30, 60, 120 and 240 min of incubation with itraconazole respectively (Fig. 1). A volcano plot for each time point was created to assess the distribution of the log_2_FC values relative to the corrected *p*-value (Fig. 2).

### Verification of RNA-seq data by qRT-PCR

To check the quality of our RNA-seq dataset, we verified the expression of eight differentially expressed genes (DEGs) over a range of fold changes by conducting quantitative real time PCR (qRT-PCR) experiments. Genes were selected from different categories: up-regulated, down-regulated or non-regulated. Patterns of the RNA-seq analysis were highly similar to the patterns obtained from the qRT-PCR assays, confirming that our sequencing results were reliable (Additional file 4: Figure S3).

### Functional characterization of DEGs

**Fig. 3:**
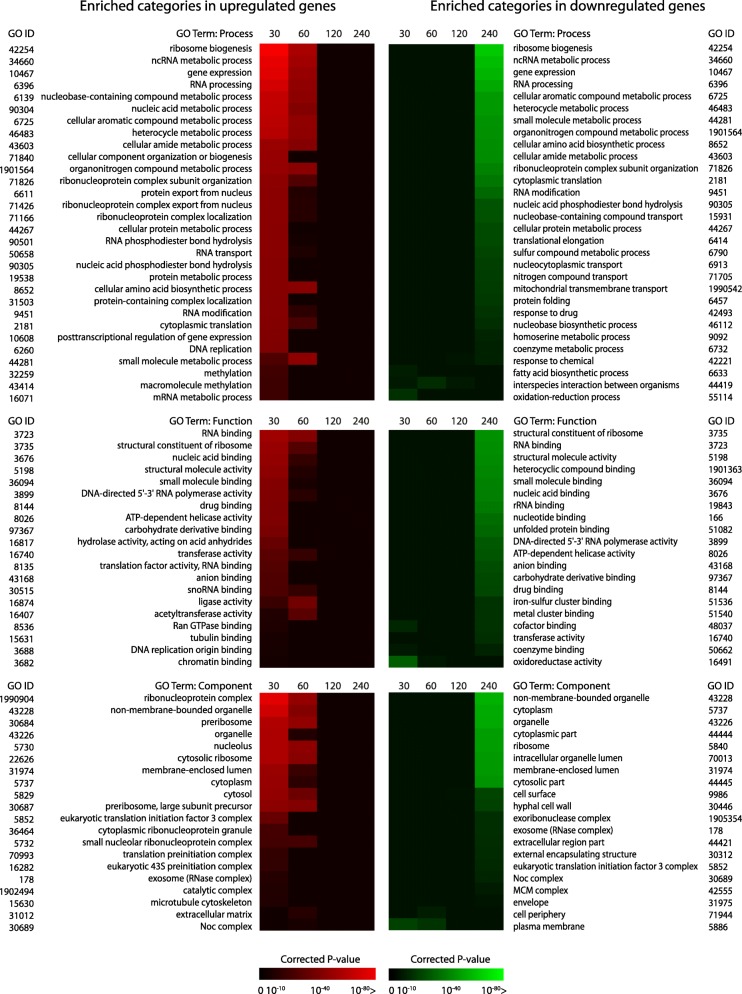
Heatmap of the most significant GO terms in the categories biological process, molecular function and cellular component. Highly redundant terms were removed with REVIGO. GO term enrichment was performed for the significant up- and down-regulated genes separately, for each time point. The colors represent the corrected p-value of each term

To investigate whether itraconazole-responsive genes could be functionally categorized, Gene Ontology (GO) enrichment analysis was performed using the AspGD GO term finder. After removal of general (e.g. ‘biological process’, ‘metabolic process’) and redundant categories with REVIGO [33], the most enriched terms from the categories Biological Process, Molecular Function and Cellular Component, were selected respectively. GO term analysis was performed for both the up-regulated and down-regulated genes [34] (Fig. 3). In general, after 30 min of incubation with itraconazole, the overall results showed a large group of enriched categories in the up-regulated genes and a small group of enriched categories of down-regulated genes. After 240 min, these numbers were reversed; a large group of enriched categories in the down-regulated genes and a small group of enriched categories was found in the up-regulated genes. Overall, no enriched terms were found in the up-regulated genes after 120 and 240 min.

Of the 30 most enriched GO-terms in the Biological process category, the terms ‘ribosome biogenesis’, ‘ncRNA metabolic process’, ‘gene expression’ and ‘RNA processing’ were most significant in the up-regulated genes after 30 min, and in the down-regulated genes after 240 min. In addition, most enriched processes in the up-regulated genes were related to amino acid metabolism, nucleic acid metabolism, transcription, translation and general transport of RNA and proteins. The only processes enriched in the down-regulated genes after 30 and 60 min were ‘fatty acid biosynthetic process’, ‘interspecies interaction between organisms’ and ‘oxidation-reduction process’. These results indicate a decreased production of fatty acids during the first 60 min. Furthermore, the enrichment of ‘interspecies interaction between organisms’ indicates a decreased expression of genes involved in, for instance, host interaction.

After 240 min, many terms were enriched in the down-regulated genes, including ‘response to drug’ and ‘response to chemical’, indicating that transcription of genes that was altered as a response to the addition of itraconazole, is especially important during the first 2 h of incubation.

In the category Molecular Function, the term ‘drug binding’ was enriched in the up-regulated genes after 30 min and enriched in the down-regulated genes after 240 min, indicating the increase of gene products that bind specifically to a drug during the first 30 min. Furthermore, ‘unfolded protein binding’, was enriched in the down-regulated genes after 240 min. This term mainly refers to binding unfolded ER proteins, indicating a possible increase of incorrectly folded proteins that activate an ER-stress response.

In the Component category, various terms specifically referred to adjustments to the exterior of the cell. As itraconazole disturbs ergosterol biosynthesis, it is likely that membrane integrity is impaired. Interestingly, the cell adjusts various transcriptional processes related to the plasma membrane and the fungal cell wall, as a response to itraconazole. The GO term ‘plasma membrane’ was the only enriched term after 30 min in the down-regulated genes. After 60 min, the term ‘cell periphery’ was also enriched in the down-regulated genes, and the terms ‘hyphal cell wall’ and ‘cell surface’ were enriched in the down-regulated genes after 240 min.

### DEGs during all time points

As the majority of all genes is differentially expressed only transiently, it would be valuable to assess whether certain genes would be differentially expressed during the whole time course, indicating an important role for these genes in the transcriptional response to itraconazole.

**Fig. 4:**
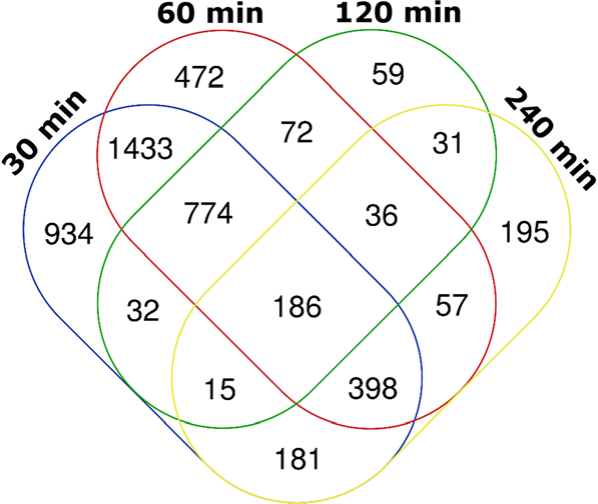
Venn diagram showing the overlap between the differentially expressed genes on each time point. A core set of 186 genes was differentially expressed during all time points

We investigated all genes that were differentially expressed during all time points, and a core group of 186 genes was identified (Fig. 4). Down-regulated genes included for instance; the bZIP transcription factor Yap1 (Afu6g09930), the sensor histidine kinase Fos-1/TcsA (Afu6g10240), the major allergen Aspf2 (Afu4g09580), and the putative calcineurin binding protein CbpA (Afu2g13060). Amongst the constant up-regulated genes were the putative sterol 14α demethylase Erg6 (Afu4g03630) and the fatty acid oxygenase PpoA (Afu4g10770).

Furthermore, nine transcription factors, three RING finger proteins, five heat shock proteins, six MFS transporters and 26 hypothetical proteins with no current known function were identified.

A complete overview of all differentially expressed core group genes is shown in Additional file 5: Table S2.

### Analysis of the top DEGs

**Fig. 5:**
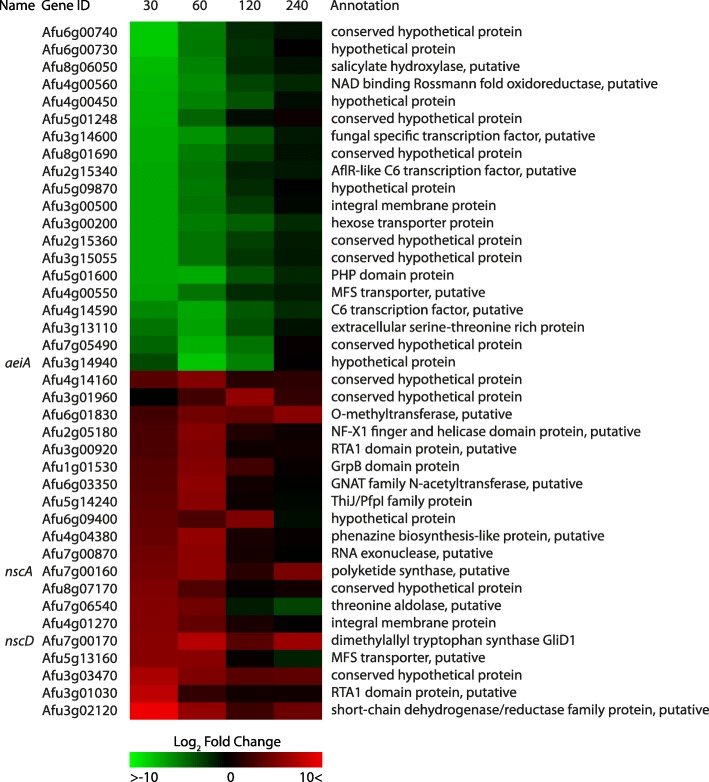
Heatmap of the 40 top up-and down-regulated genes. The colors represent the Log2FC of each time point, relative to reference time point *t* = 0

We investigated which individual genes have the highest changes in gene expression, to assess which genes play an important role in the physical response to the addition of itraconazole to the cell. Figure 5 shows a heatmap of the 20 top up-regulated and 20 top down-regulated genes. Of these 40 genes, 14 genes were annotated as ‘hypothetical protein’ or ‘conserved hypothetical protein’. The putative elastase-inhibitor *aeiA* (Afu3g14940) showed the most down-regulation after 60 min. Furthermore, three putative transcription factors; ‘fungal specific transcription factor’ Afu3g14600, ‘AflR-like C6 transcription factor’ Afu2g15340 and C6 transcription factor Afu4g14590 were strongly down-regulated after 30 to 60 min. Among the 20 most up-regulated genes, the ‘short-chain dehydrogenase/reductase protein’ Afu3g02120 was the most up-regulated after 30 min. The two putative RTA1 domain proteins Afu3g00920 and Afu3g01030 showed a strong up-regulation after 30 to 60 min. Interestingly, *nscA* (Afu7g00160) and *nscD* (Afu7g00170) which are both encoded in the nsc/fcc secondary metabolite gene cluster, were strongly up-regulated. These genes are implicated to play a role in neosartoricin and fumicycline A biosynthesis. Neosartoricin was previously identified as an immunosuppressive polyketide, and fumicycline A production was shown to be induced upon exposure to the bacterium *Streptomyces rapamycinicus*, although no further functions have been addressed to this compound [35, 36].

### Expression profile of genes encoding putative membrane transporters

**Fig. 6:**
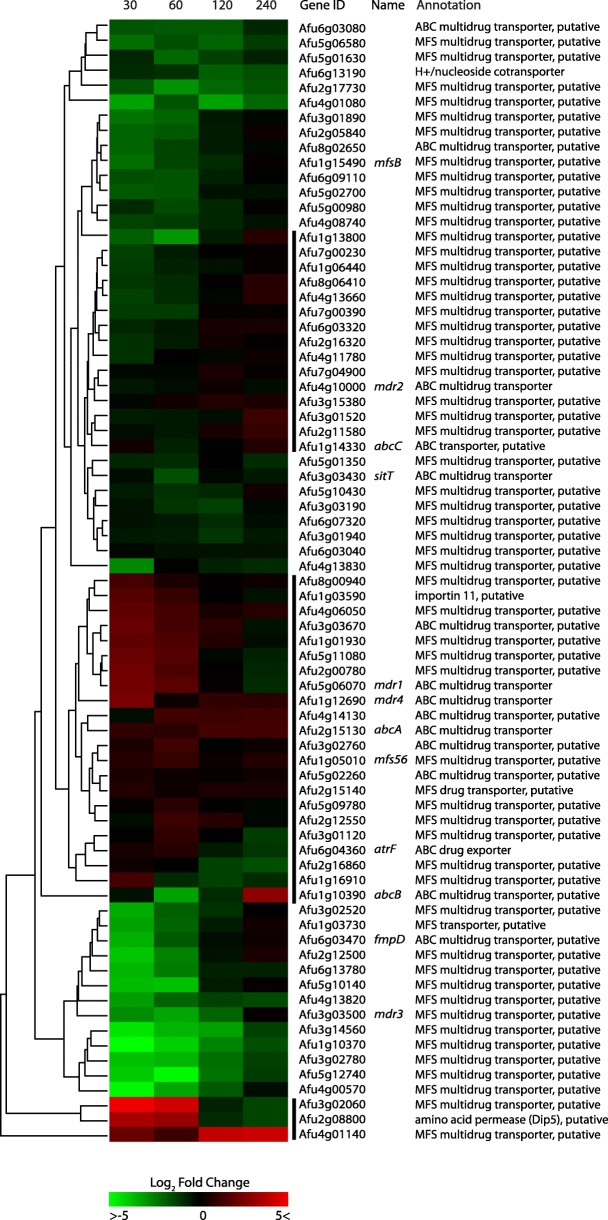
Heatmap showing the hierarchical clustering of the expression patterns of all transporters predicted to play a role in drug-efflux in *A. fumigatus*. The colors represent the Log2FC of each time point, relative to reference time point *t* = 0. Euclidean distances were calculated and complete-linkage hierarchical clustering was performed. The four bars indicate groups that show moderate to strong up-regulation and could play an important role in itraconazole-efflux

Many studies have emphasized the important role of drug efflux transporters in drug resistance in various fungal species. These transporters can be categorized as an ATP-binding cassette transporter (ABC-transporter) or a Major Facilitator Superfamily transporter (MFS-transporter), and play an important role in fungal survival [37–39]. To investigate their role in PP a high-throughput screen is necessary. We have investigated 75 DEGs involved in or predicted to be involved in drug efflux and performed hierarchical clustering based on their expression pattern (Fig. 6). Amongst the several verified multidrug efflux transporters, *mdr1* (Afu5g06070) and *mdr4* (Afu1g12690) were found to be the most up-regulated during the first 30 to 60 min, additionally *abcA* (Afu2g15130) showed an increased expression during all time points measured. Other up-regulated verified transporters include *abcB (*Afu1g10390), *abcC* (Afu1g14330) and *mfs56* (Afu1g05010). Furthermore, among the other drug-efflux transporters, 25 putative transporters in three different clusters indicated by a black sidebar, showed a significant up-regulation and a similar pattern to the known drug-efflux transporters. These transporters are implicated to play a role in drug-efflux and should be investigated.

### The effect of itraconazole on membrane homeostasis

Azole compounds interfere with ergosterol biosynthesis, which is suggested to have a significant impact on membrane rigidity [40]. Therefore, we analyzed all genes involved in the ergosterol biosynthesis pathway. Additionally, we analyzed the key pathways involved in phospholipid biosynthesis; the CDP-DAG pathway and the Kennedy pathway, which are postulated to be very similar to the *Saccharomyces cerevisiae* pathways [41]. These pathways are involved in the production of phospholipids from phosphatidic acid de novo, and the production of phosphatidylethanolamine (PE) and phosphatidylcholine (PC) from exogenous ethanolamine and choline, respectively [42]. Therefore, they play an important role in maintaining membrane integrity and DEGs in these pathways could provide leads on how *A. fumigatus* exerts its phenotypic plasticity.

### Differentially expressed genes in the ergosterol biosynthesis pathway

**Fig. 7:**
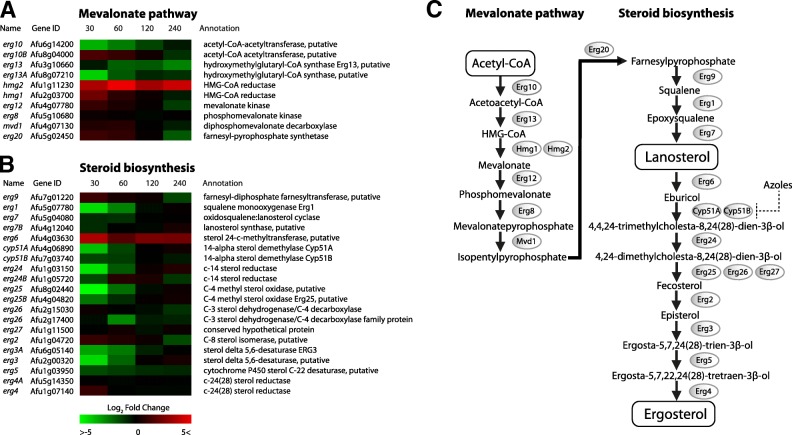
The colors represent the Log2FC of each time point, relative to reference time point *t* = 0. **a** Heatmap of all enzymes with a verified or predicted role in the mevalonate biosynthesis. **b** Heatmap of all enzymes with a verified or predicted role in the ergosterol biosynthesis. The *erg6* gene shows strong up-regulation through the entire time series. **c** Mevalonate and ergosterol biosynthesis pathways in *A. fumigatus*

To investigate the transcriptional effects of Cyp51A inhibition in the ergosterol biosynthesis pathway, the expression profile of 30 genes involved in ergosterol biosynthesis was analyzed, including those in the mevalonate pathway (Fig. 7a). The mevalonate pathway facilitates the conversion of acetyl-CoA to farnesylpyrophosphate, the first compound utilized in the ergosterol biosynthesis pathway. The *erg13A* gene showed the highest down-regulation of log2FC -4.2 after 30 min, while the hydroxymethylglutaryl-CoA (HMG-CoA) reductase *hmg1* was the most up-regulated. The other putative HMG-CoA reductase *hmg2* showed a strong up-regulation, but was not considered significantly differentially expressed due to an average read count of 2.53 in the 30 sequenced samples.

In the steroid biosynthesis pathway, an overall down-regulation of genes within 30 min was observed, including down-regulation of *cyp51A* and *cyp51B* (Fig. 7b). Out of the 20 genes, 15 genes were found to be down-regulated. The strongest up-regulation at all time points was measured at the *erg6* gene, which functions directly upstream of Cyp51A and Cyp51B. The other genes that were measured to be up-regulated, were the C-8 sterol isomerase gene *erg2* and the C-24 sterol reductase *erg4.*

### Phosphatidylethanolamine biosynthetic genes show fast up-regulation after 30 min

**Fig. 8:**
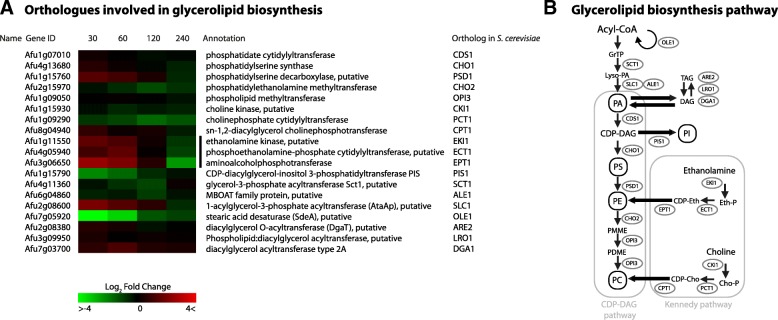
**a** Heatmap of all *A. fumigatus* enzymes homologous to *S. cerevisiae* enzymes which exert a key function in (phospho)lipid metabolism. The colors represent the Log2FC of each time point, relative to reference time point t = 0. The genes indicated by a black sidebar are orthologues to the *S. cerevisiae* genes responsible for de novo biosynthesis of phosphatidylethanolamine, and show a 1.5–2.4 log2FC. **b** Glycerolipid biosynthesis pathways in *S. cerevisiae*

Analysis of lipid biosynthetic genes showed mainly up-regulation of genes involved in the production of one of the major phospholipids, phosphatidylethanolamine [43] (Fig. 8). The mitochondrial phosphatidylserine decarboxylase *psd1* was up-regulated log2FC 1.36 after 30 min. Furthermore, the genes involved in the production of PE from extracellular ethanolamine were up-regulated after 30 min; *eki1*, *ect1* and *ept1* showed an up-regulation of log2FC 1.49, 1.52 and 2.54, respectively. After 240 min, these genes were down-regulated. The most down-regulated genes included the C16:0-CoA desaturase *ole1* with a log2FC of − 4.16, and the CDP-diacylglycerol-inositol 3-phosphatidyltransferase *pis1* with a log2FC of − 2.22 after 30 min. Down-regulation of the acyl-CoA desaturase *ole1* will decrease the synthesis of unsaturated fatty acids [44], increasing membrane lipid saturation. An increase in membrane lipid saturation increases membrane rigidity [45], possibly as a mechanism to compensate for the loss of rigidity due to ergosterol depletion.

### Differential expression of MAP kinases

**Fig. 9:**
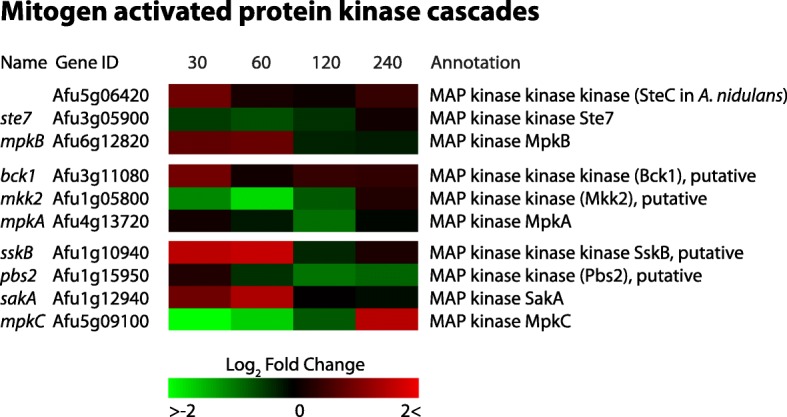
Heatmap of all verified and putative MAP kinases in *A. fumigatus*. The colors represent the Log2FC of each time point, relative to reference time point t = 0. The MAPK *sakA* and the MAPKKK *sskB* show a 1.3 log2FC and a 1.5 log2FC after 60 min, respectively

Previous research showed that Hsp90 and MAPK proteins are involved in the azole-induced response to membrane stress in *Candida spp.* [46]. To investigate which signaling pathways could be potentially involved in the physical response to itraconazole, we investigated the MAPK cascades present in *A. fumigatus* [31, 47]*.* Ten genes have been identified as potential components of three MAPK cascades in *A. fumigatus* (Fig. 9) [31]. The MAPKK Mkk2 (Afu1g05800) is homologous to *S. cerevisiae* ScMKK2, and is involved in cell wall integrity signaling and membrane fluidity sensing [48, 49]. We observed that *mkk2* was down-regulated log2FC of − 1.61 after 60 min. The proteins MAPKKK SskB (Afu1g10940), MAPKK Pbs2 (Afu1g15950), MAPK SakA (Afu1g12940) and MpkC (Afu5g09100) have been identified to play a role in high osmolarity signaling [50, 51], and are considered homologs of the HOG-MAPK pathway in *S. cerevisiae*. The *sskB* gene was up-regulated log2FC 1.45 after 60 min. The MAPK proteins SakA and MpkC were up-regulated log2FC 1.26 after 60 min, and up-regulated log2FC 1.33 after 240 min, respectively. Additionally, we found that homologs of three genes involved in glycerol formation in *A. nidulans* were up-regulated [52, 53]. The putative glycerol-3-phosphate dehydrogenase *gfdA* (Afu1g02150) and the putative glycerol-3-phosphate phosphatase *gppA* (Afu1g10570) showed up-regulation of log2FC 1.86 and 1.24 after 30 min, respectively. The other putative glycerol-3-phosphate dehydrogenase *gfdB* (Afu2g08250) showed up-regulation of log2FC 1.38 after 60 min (Additional file 6: Table S3).

### Comparison with transcriptome data of several stresses

**Fig. 10:**
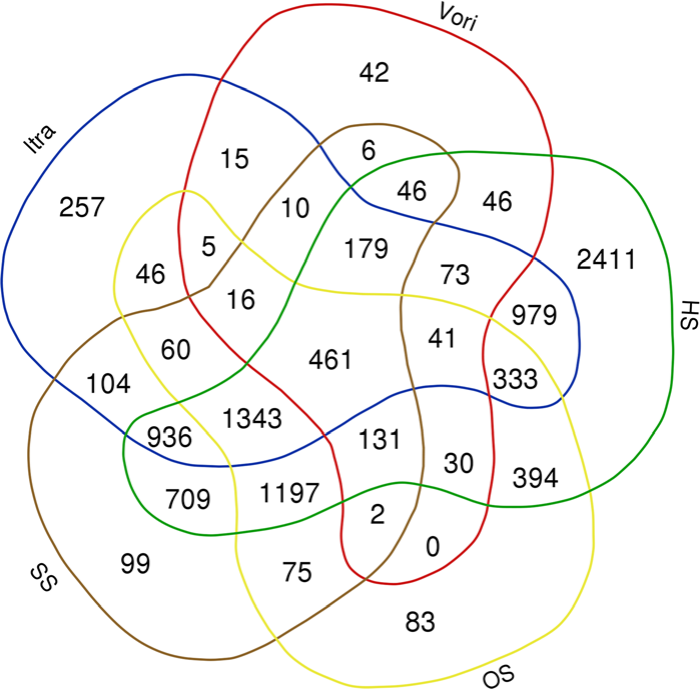
Itraconazole responsive genes. Venn diagram comparing all DEGs of this study and the DEGs found by da Silva Ferreira et al., and Takahashi et al.*,* after exposing *A. fumigatus* to different stresses to compare transcriptional responses. After removal of merged genes, 251 unique DEGs remain which were only differentially expressed upon exposure to itraconazole

In this study, we have described the general transcriptional patterns at four time points, various categories of genes and several groups of genes in particular, in the response of *A. fumigatus* to itraconazole. It should be noted that many transcriptional changes measured are presumably part of a general stress response which is activated not only in response to itraconazole stress. To investigate this, all differentially expressed genes were compared with the transcriptome data of previously conducted research [47, 54]. In the study of Da Silva Ferreira et al. mycelia were exposed to voriconazole, and analyzed after 30, 60, 120 and 240 min of incubation via microarray analysis. In the study of Takahashi et al.*,* various stress responses were induced through heat shock (37 °C and 48 °C), osmotic stress, and superoxide stress, and the transcriptomic changes were monitored through RNA-seq at 15, 30, 60, 120 and 180 min after induction. The genes were selected based on the cutoff values log2FC > 1 or < − 1, and DEGs at all time points were combined. DEGs from both heat shock treatments were also combined. A Venn diagram overlapping these genes was generated, (Fig. 10, Additional file 7: Table S4). A group of 257 genes was identified uniquely to be differentially expressed upon exposure to itraconazole (Additional file 8: Table S5). Of these 257 unique genes, five genes were merged due to complex annotation changes. The most up-regulated gene during all time points with a log2FC 2.7 to 10.7 has predicted oxidoreductase activity and a role in metabolic process (Afu3g02120). The most down-regulated gene after 30 min with a log2FC of − 7.6 has predicted oxidoreductase activity and a role in an oxidation-reduction process (Afu4g00560). The most down-regulated gene after 60, 120 and 240 min with a log2FC of − 4.1 to − 6.1 has predicted coenzyme binding and oxidoreductase activity (Afu5g01250). Several other down-regulated genes in this group are the O-methyltransferase *tpcA* (Afu4g14580), the putative alpha-amylase *amyA* (Afu3g00900), and the essential 1,3-beta-glucanosyltransferase *gel4* (Afu2g05340). Additionally, the putative C-5 sterol desaturase *erg3C* (Afu8g01070), the putative sterol sensing factor *insA* (Afu4g07680), and an ortholog of a gene which exhibits intermembrane sterol transfer activity in *S. cerevisiae* (Afu5g07100), are down-regulated. The up-regulated genes contain the allergen *aspf28* (Afu6g10300), the parvulin-like peptidyl-prolyl cis-trans isomerase *par1* (Afu1g05450), and the *cbkA* gene (Afu1g09950) which is regulated by the *crzA* transcription factor in *A. fumigatus* [55].

## Discussion

The goal of this study was to investigate differential gene expression in azole-susceptible clinical strains after the addition of sub-lethal concentrations of itraconazole, to gain valuable insights into the way *A. fumigatus* exerts its phenotypic plasticity and manages to survive. We gained insight in the general expression patterns which take place as *A. fumigatus* adapts to the presence of an antifungal compound, and identified 25 potential drug-efflux transporters which potentially aid the fungus to survive by exporting itraconazole from the cell. Furthermore, we demonstrated that the *erg6* gene was up-regulated during the whole time course and that the genes facilitating PE production from extracellular ethanolamine were up-regulated, indicating an increased production of this phospholipid. Finally, we observed an increased expression of several MAPK cascade proteins which are also involved in HOG signaling. This study is, to our knowledge, the first study to use RNA-seq to measure gene expression of clinical isolates in response to itraconazole.

In our experimental setup, we compared the isolates of each time point with the isolates directly harvested after 24 h. Although we do not distinguish between the transcriptional changes caused by itraconazole or other changes related to mycelial growth progression, previous research demonstrated that *A. fumigatus* resides in the exponential growth phase after 24 h, in various nutrient media [56]. Interpreting transcriptomics results is limited by the fact that only 487 (4.95%) genes have been experimentally verified and characterized in *A. fumigatus*, making the interpretation of transcriptomics results challenging (*Aspergillus fumigatus Af293* Genome Snapshot, AspGD, May 14th, 2018). Although many gene functions are predicted computationally through known functions of homologous genes in other closely related species, they are yet to be experimentally confirmed. It was notable that the number of enriched GO terms per time point was not a direct consequence of the number of genes in the group; the 2254 down-regulated genes after 30 min yielded only five enriched GO terms in the category process, as opposed to the 1701 up-regulated genes after 30 min, which yielded a total of 194 enriched GO terms in the category process before the removal of redundant terms. This suggests that many cellular processes are down-regulated after 30 min, but all to similar extent as no specific terms are enriched.

GO term enrichment analysis showed increased activity of various specific processes related to the exterior of the cell, consistent with previous research that states that itraconazole decreases membrane integrity by interfering with ergosterol biosynthesis [40, 57]. The cellular component category also showed the enriched term ‘extracellular matrix’ in the up-regulated genes after 60 min, which mainly comprises of genes which are part of the fungal biofilm matrix. These biofilms are often involved in host adherence and provide protection from extracellular stresses, suggesting an important role for biofilm formation in the physiological response of *A. fumigatus* [58, 59].

The involvement of elastase in fungal virulence is discussed in several studies, and its contribution to pathogenicity, by breaking down elastin in lung tissue, is not well established for *A. fumigatus* [60–63]. We found that the putative elastase inhibitor *aeiA* (Afu3g14940) was the most down-regulated gene in our dataset (log2FC 8.27). There is a possibility that these clinical strains have specifically adapted to the lung environment, and that there are other beneficial effects that do not influence virulence, although these results suggest that *A. fumigatus* greatly adjusts its elastase activity upon itraconazole stress.

Amongst the strongest up-regulated genes were two putative RTA1 domain proteins. Not much is known about this gene family, that was initially found to be involved in the export of long sphingoid bases in *S. cerevisiae* [64]. Interestingly, overexpression of ScRTA1 results in 7-aminocholesterol resistance, an antifungal compound that inhibits sterol biosynthesis. Heme-deficiency was also shown to induce ScRTA1 expression. It is likely that disturbance of ergosterol biosynthesis influences iron homeostasis in the fungal cell, as these processes share mevalonate as a precursor and are both regulated by the sterol regulatory element binding protein SrbA [65, 66]. When heme or iron sources are deficient, siderophore mediated iron-uptake is increased, and *hmg1* expression is up-regulated. This is consistent with our finding that *hmg1* expression is increased [64, 66]. The role of RTA1 proteins in the physical response to azoles has not yet been established and could provide an interesting lead.

A large number of transporters have been described in *A. fumigatus,* and the fungus can defend itself against a large number of toxic compounds via for instance drug-efflux pump activity [67, 68]. The genome of *A. fumigatus* is predicted to contain at least 49 ATP binding cassette (ABC) family transporter genes and 278 major facilitator superfamily (MFS) genes. From the AspGD website, only genes encoding transporters were extracted that were predicted to play a role in drug efflux specifically. This might leave out potential other transporter genes, which are not yet computationally predicted to be involved in drug efflux in general. Nevertheless, 25 transporters showed comparable expression patterns to the verified drug-efflux transporters within the first. These transporters could play an important role in physical resilience induced by PP, and their involvement should be addressed experimentally in future research.

We investigated to what extent itraconazole influences the expression of ergosterol biosynthetic genes. Alterations in the ergosterol biosynthesis pathway have been shown to confer resistance in a previous study with *Candida albicans* [69]. Strikingly, *erg6* showed a significant up-regulation of log2FC 1.78–3.54 during the whole time course, as opposed to the rest of the ergosterol biosynthetic pathway, which showed a general down-regulation, or no-regulation. Recent studies suggested that not Cyp51A, but Erg6 is the 14α-lanosterol demethylase in *A. fumigatus,* as opposed to *S. cerevisiae,* where ScERG*6* encodes a sterol 24-C-methyltransferase further downstream [8, 70–72]. Erg6 functions directly upstream of Cyp51A, where it first converts lanosterol to eburicol. Inhibition of Cyp51A by azoles and up-regulation of *erg6* would then lead to a strong accumulation of eburicol. Why the increased production of eburicol would be a favorable trait when this compound possibly already accumulates as the Cyp51A enzyme is inhibited by azoles [8], is not clear. It is possible that a feedback regulation mechanism senses the absence of the Cyp51A/B product, 4,4,24-trimethylcholesta-8,24(28)-dien-3β-ol, when they are inhibited by azoles with the increased production of their substrate as a direct result.

The genes involved in biosynthesis of phosphatidylethanolamine (PE) from extracellular ethanolamine showed a predominant up-regulation. A recent study on mammalian cells suggests that PE is the key phospholipid in maintaining proper membrane rigidity, and they found that a decrease in cholesterol resulted in a strong increase of PE relative to the other phospholipids [73]. These genes were identified as the most up-regulated of all lipid biosynthesis genes investigated in this study and could imply an increase of PE in the membranes to maintain proper rigidity, when ergosterol levels are depleted [45, 74].

The important role of cell signaling in fungal adaptation to stress factors and antifungals has been extensively demonstrated [31, 75–77]. We have investigated the involvement of MAPK cascade proteins upon azole stress. The *mpkB* gene is still uncharacterized in *A. fumigatus*, however, the *mpkB* gene in *A. nidulans* and the FUS3 gene in *S. cerevisiae* are well-described homologs. Their role in the cellular pheromone response and sexual development is established [78, 79], indicating a possible role of these processes in the response to itraconazole stress. Furthermore, we measured a significant up-regulation in the *sakA* MAPK gene, which is homologous to the HOG1 gene of *S. cerevisiae*. This gene is the key MAP kinase involved in the regulatory processes in response to osmotic, oxidative, heat, and hypoxia mediated stresses [31]. In *A. fumigatus*, *sakA* has been shown to play an important role in the osmotic and oxidative stress responses. Deletion mutants showed an increased sensitivity to itraconazole and amphotericin B [80], which is consistent with our finding that *sakA* expression is increased as a response of *A. fumigatus* to itraconazole. Our results suggest that upon exposure to itraconazole, *A. fumigatus* differentially regulates genes which are related to the osmotic stress response and increases its glycerol production. This is supported by our findings that at least three genes involved in glycerol formation were up-regulated within 60 min. Transcriptional changes of MAP kinases could give insight into the stress-response mechanisms [47], although MAP kinases are activated by phosphorylation [81]. Therefore, analysis of the phosphoproteome could provide additional insights into their direct involvement in adaptation to itraconazole stress.

Recently, the mitochondrial complex I has been implicated to play a role in azole resistance. Interestingly, our results show only a slight down-regulation of the 29.9 KD subunit of mitochondrial complex I (Afu2g10600: Log2FC -0.81, *P*-value 0.01, adjusted *P*-value 0.07) (Additional file 7: Table S4) after 240 min, although deletion of this gene leads to partial azole resistance (80). The authors suggest a resistance mechanism based on restoring an unbalanced oxidative stress response when itraconazole (1 mg/L) is added to the medium. However, the DEGs in our dataset do not indicate an oxidative response in our isolates exposed to itraconazole, as we did not measure up-regulation of genes that are induced upon hypoxic conditions [82]. Therefore, a down-regulation of the 29.9 KD subunit would not be necessary to rebalance the oxidative stress response. It is also possible that this gene is not differentially regulated and does not contribute to PP to gain azole resilience, although further research is necessary to elucidate if higher concentrations of itraconazole could result in a different expression pattern.

Many mechanisms that cope with cell stress respond to various stressful conditions, and are not limited to a specific stress condition or a specific antifungal compound [47, 83, 84]. To investigate if *A. fumigatus* differentially expresses genes solely in response to itraconazole, we compared our results to the transcriptomics data of previously conducted studies [47, 54]. We found that 251 genes were differentially regulated specifically in response to itraconazole. Although genetic and physiological differences between isolates compared across different studies may influence this outcome, these results suggest that transcription of these genes is specifically altered as a response to itraconazole stress. Amongst these genes is the down-regulated *insA* (Afu4g07680) gene, which is a putative sterol sensing factor. The mammalian homolog encodes an ER-anchored protein which prevents the transcriptional activation of genes involved in the synthesis and uptake of cholesterol, by binding to sterol regulatory element binding proteins (SREBPs) [85]. It is possible that *A. fumigatus* anticipates on ergosterol deficiency by down-regulating this gene to prevent inhibition of sterol synthesis and uptake, although little is known about the sterol sensing mechanisms in *A. fumigatus*.

Remarkably, previous reported DEGs resulting from voriconazole treatment share but 72,6% with DEGs from our study, despite of their shared mechanism of action. These difference could be appointed to the treatment of the isolates with a higher concentration of voriconazole (0.5 μg/mL) and although the exact MIC of the tested isolates was not documented, this could explain the different DEGs found. Additionally, the isolates tested in this study demonstrated that even between different isolates that have the same MIC, the basal expression of the transcriptome can differ. Furthermore, the difference could be appointed to the comparison of microarray results with RNA-seq results, with the latter having higher resolution. Future studies could be improved by introduction of a conserved laboratory strain in every experiment, which would facilitate better comparison.

As demonstrated in this study, RNA-seq is a promising method to provide better insight in the physiological changes and cellular defense mechanisms [86–88]. Although many processes can be monitored this way, it should be taken into account that a decreased or increased transcription, does not necessarily lead to an increased translation. Additionally, post-translational processes could further impact on the suggested increased abundance of activated proteins. It is essential that potential genes or pathways involved in the cellular response to itraconazole are confirmed by the generation of gene-deletion mutants. Furthermore, it could be promising to look at the direct consequences of the various transcriptional changes related to ergosterol and lipid biosynthesis, by investigating the full lipidome of *A. fumigatus* mycelia under various conditions.

## Conclusions

Our data strongly suggests that phenotypic plasticity allows *A. fumigatus* to substantially adapt its transcriptome within 60 min of exposure to itraconazole, after which the cells proposedly reach an improved homeostatic state. We showed that short-term adaptation through PP includes immediate up-regulation of several drug-efflux membrane transporters, activation of signaling cascades, and various alterations in gene expression in the ergosterol biosynthesis and phospholipid biosynthetic processes. Thus, our study supports the notion that transcriptomic analyses by RNA-seq can give major insights in the differentially expressed genes, enabling us to understand how the direct response of *A. fumigatus* to itraconazole promotes survival of the fungus in the patient and in the outside environment before any stable genetic mutations arise. Further research on PP and the short-term adaptation processes of *A. fumigatus* can improve the understanding on how the fungus survives during azole exposure. Increasing this fundamental knowledge is crucial in managing the spread of azole resistant *A. fumigatus* isolates and developing future antifungal therapies.

## Methods

### Isolates, media and culture conditions

Isolates were used with an MIC of 0.5 or 1 for itraconazole and the same MIC for voriconazole and posaconazole respectively, to exclude the possibility that great differences in outcome could be assigned to the differences in MICs for these isolates. The isolates were selected from three different patients from separate hospitals in the Netherlands. All isolates are listed in Table 1. Isolates were cultured on either Sabouraud Dextrose Agar (SDA) (Oxoid) (1% peptone, 4% glucose, 1.5% agar, pH 5.6) or *Aspergillus* Minimal Medium (AMM) as described in the supplemental material of reference [89] containing per liter: 10 g glucose, 5.95 g NaNO_3_, 0.522 g KCl, 1.5 g KH_2_PO_4_, 50 mg MgSO_4_·7H_2_O, 1 mL trace elements). Trace elements contained (per 200 mL): 10 g EDTA, 4.4 g ZnSO_4_·7H_2_O, 1.01 g MnCl_2_·4H_2_O, 0.315 g CuSO_4_·_5_H_2_O, 0.22 g (NH_4_)_6_Mo_7_O_24_·4H_2_O, 1.0 g Fe(II)SO_4_·7H_2_O, and 2.2 g H_3_BO_3_ [90]. All compounds were produced by Merck (Darmstadt, Germany). Isolates were stored in 20% glycerol at − 80 °C, and subcultured on SDA at 37 °C for 4–5 days. Conidia were harvested with a wet cotton swab and resuspended in Milli-Q containing 0.1% Tween 20. Approximately 2,6 × 10^5^ CFU/mL of conidia were used to inoculate 5 mL of AMM in 15 mL tubes. Conidia were inoculated in duplicate. After 24 h of growth at 37 °C while rotating, itraconazole (Sigma) was added at IC_50_ to each tube except the *T* = 0 tubes, which were immediately harvested through Miracloth (Merck)_._ Remaining mycelia were harvested through Miracloth after 30, 60, 120 and 240 min. Harvested mycelia were directly frozen in liquid nitrogen.

### Dose-response curves

For determining the itraconazole concentration where growth is estimated to be 50% (IC50), dose-response curves were made by growing each strain (*n* = 5) in a 96-wells plate containing AMM with itraconazole concentrations ranging from 0.016 μg/mL to 8 μg/mL. Plates were kept at 37 °C in a microtiter plate reader (Anthos Labtec Instruments GmbH, Salzburg, Austria) for 48 h, and optical density (OD) was measured every hour at 405 nm. After subtraction of the ODs of the empty wells, the percentage of growth from the ODs of the inoculated wells for each well was correlated with the relative OD estimated by the following equation: (OD_405_ of wells that contained itraconazole/OD_405_ of the itraconazole-free well) × 100. The relationship was determined by a non-linear regression analysis and the Hill equation with a variable slope fitted to the data. Goodness of fit was checked by the *r*^2^ values. All analyses were performed by using GraphPad Prism, version 6.0, for OS X (GraphPad Software, San Diego, USA).

### RNA sequencing

After harvesting, mycelia were lysed by bead-bashing using a MagNa Lyser instrument, three times for 30 s at 7000 rpm (Roche, Basel, Switzerland). RNA was extracted using TRIzol reagent (Invitrogen, Breda, the Netherlands). Pellets were resuspended in 100 μL DEPC treated water. RNA integrity was verified by agarose gel electrophoresis. Total RNA was stained with Midori Green (Nippon Genetics, Dueren, Germany) and visualized with UV light. Purity of all samples (A_260_/A_280_ ≥ 1.5 and A_260_/A_230_ ≥ 2.0) was determined using a NanoDrop 1000 (Thermo Scientific, MA, US). Pooled cDNA libraries were constructed with the TruSeq RNA Sample Prep Kit v2 (Illumina, San Diego, USA) according to manufacturer instructions. Quality of the libraries was verified with an Agilent 4200 Tapestation System (Agilent Technologies). Paired-end reads of 2 × 75 bp were generated in High Output mode on a NextSeq 500 sequencer (Illumina, San Diego, USA).

### Identification of differentially expressed genes

Reads were aligned with STAR v2.5.3A [91] against the reference genome sequence of *A. fumigatus* Ensemble CADRE 30 downloaded from EnsemblFungi. Differentially expressed genes (DEGs) were identified by DESeq2 for R by Bioconductor (BaseMean ≥30, *P*-value < 0.05, adjusted *P*-value < 0.1, log2FC > 1 or < − 1), and results were corrected for multiple testing by the Benjamini-Hochberg principle [92].

### GO analysis

Functional categories of DEGs were grouped according to the Gene Ontology (GO) term, using the GO Term Finder on the AspGD website to identify significant terms (*p* ≤ 0.05) [93]. The False Discovery Rate cutoff value was set to *p* ≤ 0.05. REVIGO was used to remove redundant GO terms [33] and allowed similarity was set to 0.5. Terms with over 30% frequency were removed to avoid general terms. Up-regulated and Down-regulated genes were screened separately for enriched categories, to improve statistical power [34].

### Validation of RNA-seq results by quantitative real-time PCR

All primers are listed in Additional file 9: Table S6. Eight genes with different expression patterns were selected, and expression was confirmed in all three strains separately. RNA isolation and cDNA synthesis were performed as described above. Quantitative real-time PCR was performed with gene-specific primers, using the actin gene (*act1)* as reference gene. PCR was performed using Roche Lightcycler 480 SYBR Green I Master according to the protocol of the manufacturer. PCR conditions were as follows: Denaturation at 95 °C for 5 min, followed by 45 cycles of denaturation at 95 °C for 10 s, primer annealing at 60 °C for 10 s, and extension at 72 °C for 10 s. Expression levels were calculated by the ΔΔC_T_-method, and normalized for actin mRNA expression levels [94]. All qRT-PCR experiments were per-formed at least in duplicates.

### Statistical analysis and figure construction

Heatmaps and volcano plots were generated by Rstudio Version 1.0.143 for OS X. For generating heatmaps, gplots v3.0.1 was used. For generating volcano plots, ggplot2 V2.2.1 was used. Exported images were assembled with Adobe Illustrator CC 2015. Venn diagrams were created with Biovenn (http://www.biovenn.nl/index.php) and the Venn tool by (http://bioinformatics.psb.ugent.be/webtools/Venn). Graphs of qRT-PCR and dose-response curve analyses were generated by using GraphPad Prism, version 6.0, for OS X (GraphPad Software, San Diego, USA). Error bars represent standard error of the mean.

## Additional files

Additional file 1: **Figure S1.** Dose-response curves of the three *A. fumigatus* strains upon exposure to increasing concentrations of itraconazole, ranging from 0.016 mg/l to 8 mg/l. (PDF 36 kb)

Additional file 2: **Table S1.** Mapping results and coverage calculations. (DOCX 79 kb)

Additional file 3: **Figure S2.** PCA plot of all samples, normalized by a regularized log transformation. (PDF 113 kb)

Additional file 4: **Figure S3.** The expression levels of eight selected genes, verified by RT-PCR. The values were normalized to the actin expression, and error bars represent standard deviation of the mean. (PDF 297kb)

Additional file 5: **Table S2.** The 186 genes found to be differentially expressed at all time points. (XLSX 59kb)

Additional file 6: **Table S3.** The DESeq2 dataset results. (XLSX 5090 kb)

Additional file 7: **Table S4.** Venn analysis results of all shared genes per group. (TXT 128 kb)

Additional file 8: **Table S5.** All 251 genes that were only differentially expressed upon itraconazole stress. (XLSX 62 kb)

Additional file 9: **Table S6.** All primers used in this study. (DOCX 49 kb)

## Abbreviations

DEGs: Differentially expressed genes
GO: Gene ontology
HOG: High osmolarity glycerol
log2FC: Log2 fold change
MAPK: Mitogen activated protein kinase
PCA: Principal component analysis
PE: Phosphatidylethanolamine
PP: Phenotypic plasticity
qRT-PCR: Quantitative real time PCR
