# Supplementary figures and images for: Phenotypic plasticity and the evolution of azole resistance in Aspergillus fumigatus; an expression profile of clinical isolates upon exposure to itraconazole
Source: BMC Genomics. 2019 Jan 9;20:28. doi: 10.1186/s12864-018-5255-z (PMC6327609; doi:10.1186/s12864-018-5255-z)

## Dose-response curves

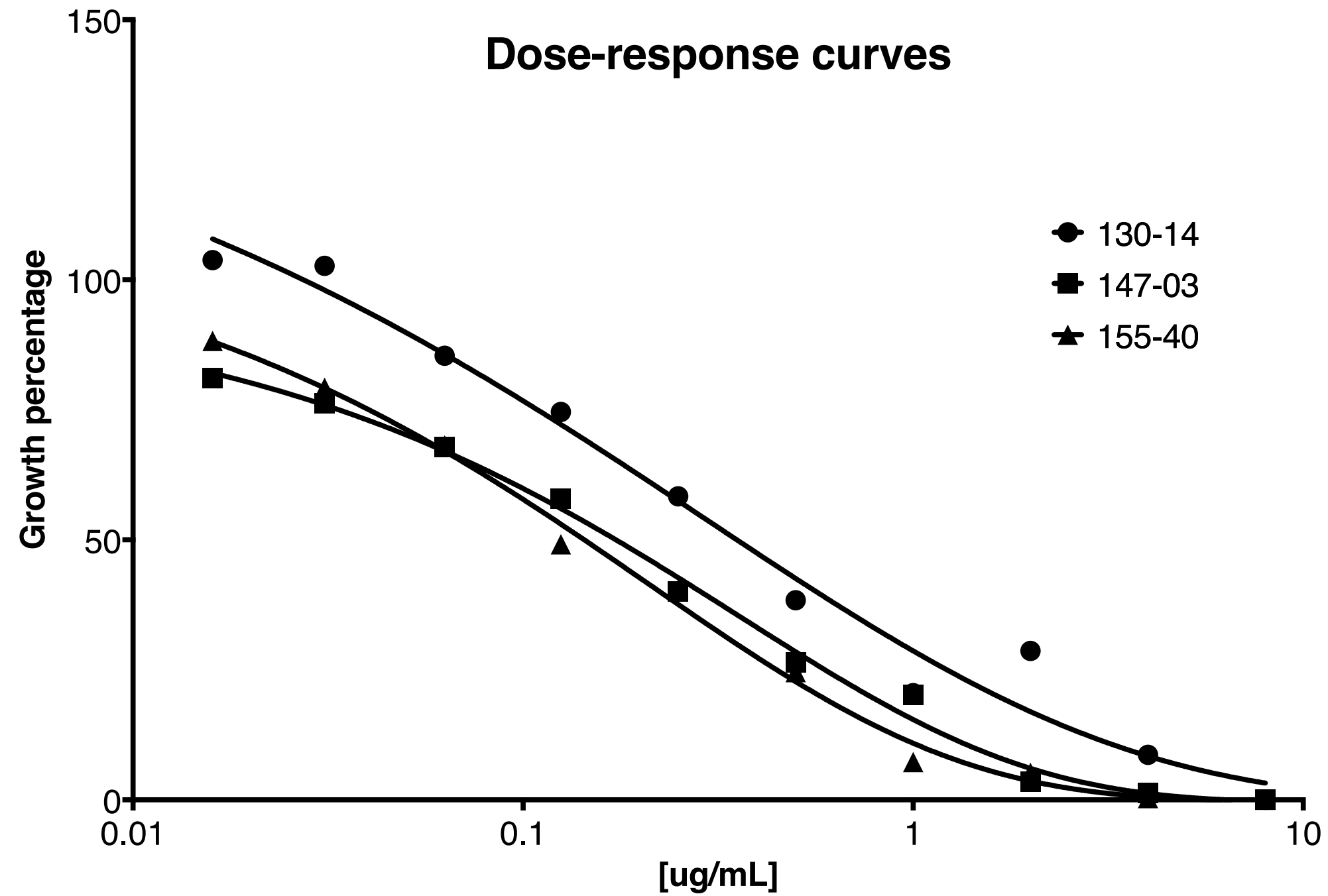

Supplement: Supplementary file 1 — Figure S1. Dose-response curves of the three A. fumigatus strains upon exposure to increasing concentrations of itraconazole, ranging from 0.016 mg/l to 8 mg/l. (PDF 36 kb) [file 12864_2018_5255_MOESM1_ESM.pdf]

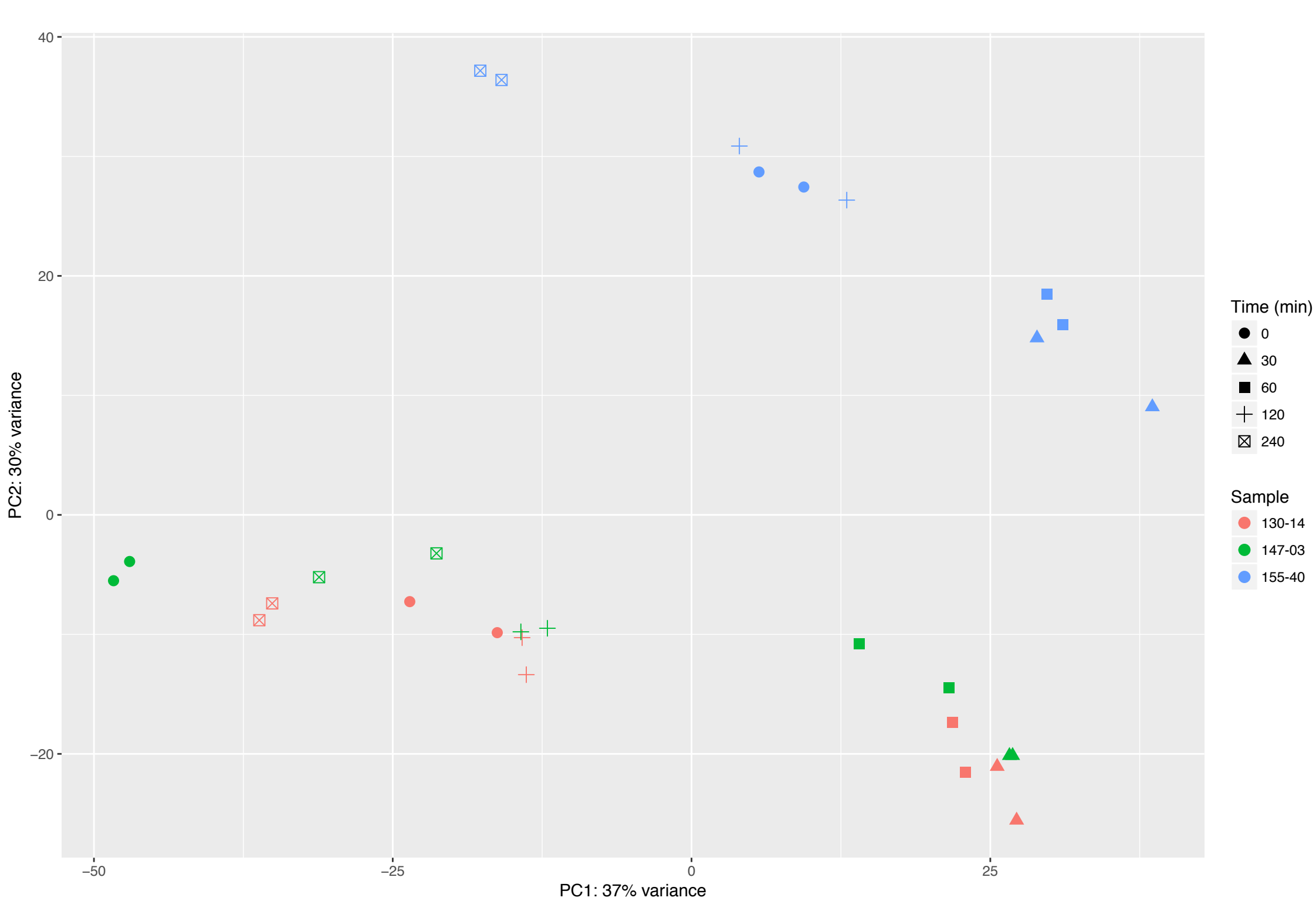

Supplement: Supplementary file 3 — Figure S2. PCA plot of all samples, normalized by a regularized log transformation. (PDF 113 kb) [file 12864_2018_5255_MOESM3_ESM.pdf]

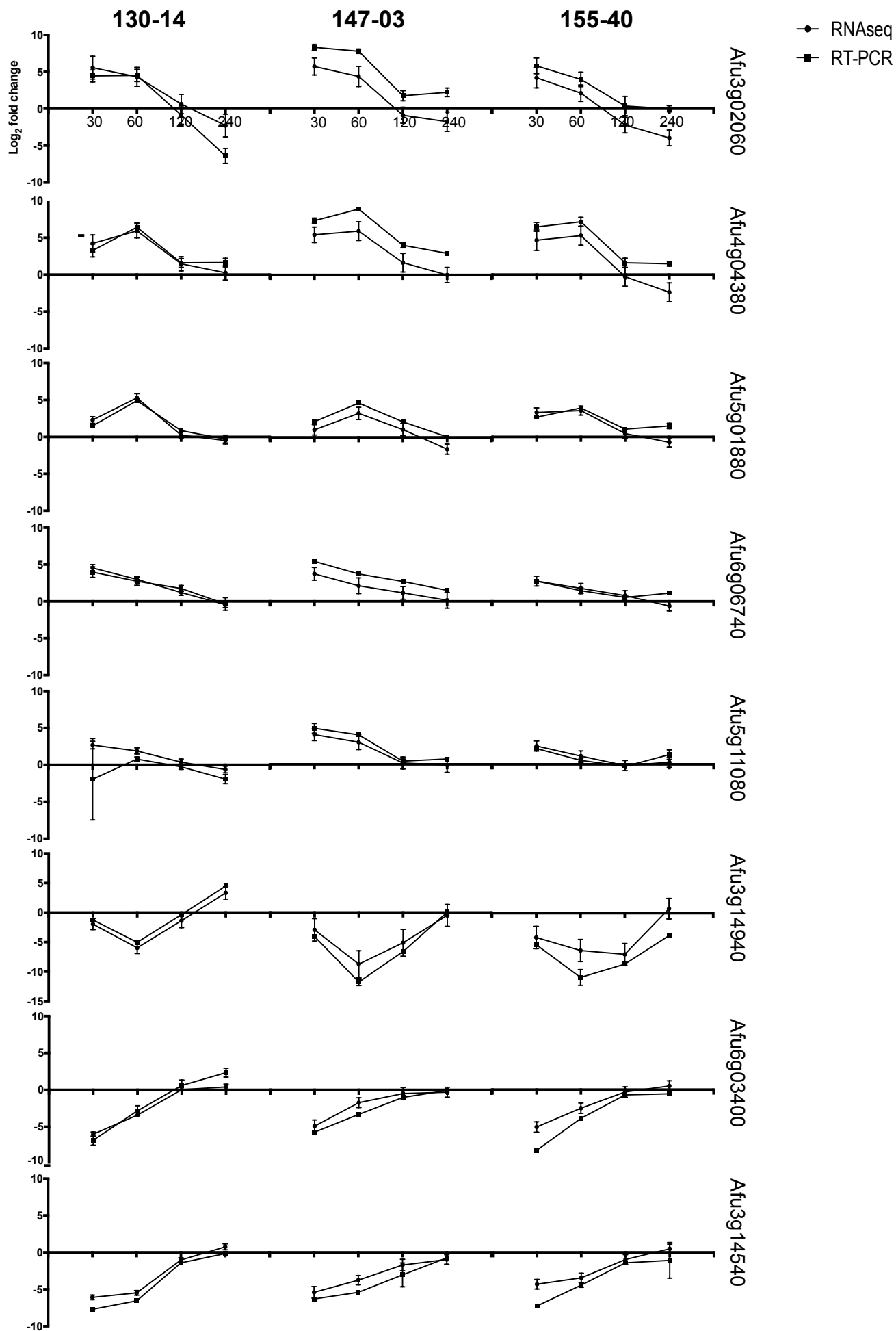

Supplement: Supplementary file 4 — Figure S3. The expression levels of eight selected genes, verified by RT-PCR. The values were normalized to the actin expression, and error bars represent standard deviation of the mean. (PDF 297kb) [file 12864_2018_5255_MOESM4_ESM.pdf]
