# Supplementary material for: Phenotypic plasticity and the evolution of azole resistance in Aspergillus fumigatus; an expression profile of clinical isolates upon exposure to itraconazole
Source: BMC Genomics. 2019 Jan 9;20:28. doi: 10.1186/s12864-018-5255-z (PMC6327609; doi:10.1186/s12864-018-5255-z)
Supplement: Supplementary file 2 — Table S1. Mapping results and coverage calculations. (DOCX 79 kb) [file 12864_2018_5255_MOESM2_ESM.docx]

| Gene ID | FW primer (5' -> 3' ) | RV primer (5' -> 3' ) | Size (bp) |
| --- | --- | --- | --- |
| Afu3g02060 | CGTAGGCGGATCGACATTCT | CCCATCGCCATGTAGTGTGT | 170 |
| Afu4g04380 | CACCATAGGAGCTGCCTGC | ACTGGTGCAGCCGTGTATTA | 170 |
| Afu5g01880 | TCATCGAGACCGGTACCAAC | CGGTGAAGGTGTCGTTCAGAT | 177 |
| Afu6g06740 | CCATGATCCGAAGAGTGGCA | CAGTGGTAGGCGTGATTCGT | 178 |
| Afu5g11080 | TTTTGGGCGCATCTCGGATA | TCATCCCCATCGAGAAAACACC | 170 |
| Afu3g14940 | CACCTGCGAAAAAGAAGCCC | TGTTCTCATTCACTTGGATGTTCA | 170 |
| Afu6g03400 | CCCAGCTTCAAAGTAGGGAGG | CGTACGCGTCCATCAGTTTT | 172 |
| Afu3g14540 | TTGCCAACAAGGACATTGAGAT | CTCTTGCTGACTTGCTTGTCC | 178 |
