# Supplementary material for: Phenotypic plasticity and the evolution of azole resistance in Aspergillus fumigatus; an expression profile of clinical isolates upon exposure to itraconazole
Source: BMC Genomics. 2019 Jan 9;20:28. doi: 10.1186/s12864-018-5255-z (PMC6327609; doi:10.1186/s12864-018-5255-z)
Supplement: Supplementary file 5 — Table S2. The 186 genes found to be differentially expressed at all time points. (XLSX 59kb) [file 12864_2018_5255_MOESM5_ESM.docx]

| Strain | Time |  | # of reads | # of uniquely mapped reads | % of mapped reads | Coverage* |
| --- | --- | --- | --- | --- | --- | --- |
| 130-14 | 0 | 1 | 7,733,135 | 7,288,085 | 94.24 | 37.94 |
|  | 0 | 2 | 4,843,518 | 4,578,451 | 94.53 | 23.83 |
|  | 30 | 1 | 13,497,421 | 12,767,704 | 94.59 | 66.47 |
|  | 30 | 2 | 19,987,386 | 18,113,064 | 90.62 | 94.30 |
|  | 60 | 1 | 20,238,170 | 17,708,376 | 87.50 | 92.19 |
|  | 60 | 2 | 6,421,402 | 4,279,251 | 66.64 | 22.28 |
|  | 120 | 1 | 13,572,239 | 12,639,563 | 93.13 | 65.80 |
|  | 120 | 2 | 3,306,780 | 2,997,040 | 90.63 | 15.60 |
|  | 240 | 1 | 7,386,321 | 6,665,525 | 90.24 | 34.70 |
|  | 240 | 2 | 5,957,106 | 5,173,822 | 86.85 | 26.93 |
| 147-03 | 0 | 1 | 33,061,799 | 30,291,634 | 91.62 | 157.71 |
|  | 0 | 2 | 19,399,232 | 17,144,287 | 88.38 | 89.26 |
|  | 30 | 1 | 26,106,386 | 23,324,601 | 89.34 | 121.44 |
|  | 30 | 2 | 27,218,933 | 25,035,131 | 91.98 | 130.34 |
|  | 60 | 1 | 4,222,887 | 3,649,084 | 86.41 | 18.99 |
|  | 60 | 2 | 3,302,484 | 3,025,432 | 91.61 | 15.75 |
|  | 120 | 1 | 18,160,364 | 15,560,484 | 85.68 | 81.01 |
|  | 120 | 2 | 27,160,844 | 24,929,956 | 91.79 | 129.79 |
|  | 240 | 1 | 8,293,798 | 7,142,264 | 86.12 | 37.18 |
|  | 240 | 2 | 6,362,191 | 5,014,607 | 78.82 | 26.10 |
| 155-40 | 0 | 1 | 14,624,928 | 13,098,358 | 89.56 | 68.19 |
|  | 0 | 2 | 7,582,874 | 6,296,720 | 83.04 | 32.78 |
|  | 30 | 1 | 8,183,483 | 6,805,838 | 83.17 | 35.43 |
|  | 30 | 2 | 6,838,761 | 3,901,393 | 57.05 | 20.31 |
|  | 60 | 1 | 8,363,749 | 7,050,698 | 84.30 | 36.70 |
|  | 60 | 2 | 7,720,845 | 6,853,901 | 88.77 | 35.68 |
|  | 120 | 1 | 22,435,728 | 20,401892 | 90.93 | 106.22 |
|  | 120 | 2 | 7,076,779 | 6,056,075 | 85.58 | 31.53 |
|  | 240 | 1 | 19,776,164 | 17,402,494 | 88.00 | 90.60 |
|  | 240 | 2 | 22,870,830 | 20,347,302 | 88.97 | 105.93 |

* Coverage is calculated as: ((# of uniquely mapped reads)*150)/28.800.000
